# The Gut Mucosal Barrier–Neuroinflammation Axis in Vascular Cognitive Impairment Induced by Chronic Cerebral Ischemia: A Narrative Review of Candidate Mechanisms

**DOI:** 10.3390/ijms27156816

**Published:** 2026-07-29

**Authors:** Jiarong Du, Lan Sun, Shanshan Wang, Yanxin Chen, Wenjuan Long, Bo Wang, Zhongyang Hu, Yujun Feng, Qiongrong Long, Mincong Huang, Xiaoya Li, Xiaoman Lv

**Affiliations:** 1 Yunnan Key Laboratory of Integrated Traditional Chinese and Western Medicine for Chronic Disease in Prevention and Treatment, Yunnan University of Chinese Medicine, Kunming 650500, China; xxr95822020@163.com (J.D.);; 2 School of Basic Medicine, Yunnan University of Chinese Medicine, Kunming 650500, China; 3 The First Clinical Medical College, Liaoning University of Traditional Chinese Medicine, Shenyang 110847, China; 4 College of Chinese Materia Medica, Yunnan University of Chinese Medicine, Kunming 650500, China

**Keywords:** vascular cognitive impairment, chronic cerebral ischemia, neuroinflammation, intestinal mucosal barrier, intestinal microflora, gut–brain axis, mechanism of action

## Abstract

Vascular cognitive impairment (VCI) is a major form of dementia with a complex pathogenesis. Emerging evidence indicates a strong interaction between intestinal mucosal barrier integrity and neuroinflammation, which has been proposed as a candidate contributor in VCI progression. This review comprehensively reviews the initiation and amplification of neuroinflammation in chronic cerebral ischemia. It highlights how impairment of the intestinal mucosal barrier, gut microbiota dysbiosis, and translocation of microbial metabolites contribute to central neuroinflammation via circulatory and neural pathways, suggesting a potential self-sustaining pathological cycle within the gut–brain axis. Furthermore, we analyze the underlying molecular mechanisms, critique current limitations, and suggest promising directions for future research.

## 1. Introduction

Vascular cognitive impairment (VCI) is a cognitive dysfunction syndrome caused by cerebrovascular disease and its risk factors (e.g., hypertension, diabetes, etc.) and covers a spectrum of diseases ranging from mild cognitive impairment to vascular dementia (VaD) [1]. As the second most common type of dementia after Alzheimer’s disease (AD), VaD accounts for about 15–20% of dementia cases worldwide [2], and its incidence continues to rise with the aging of the population, posing a serious public health challenge. The core pathological mechanism of VCI originates from neurovascular unit damage triggered by Chronic Cerebral Hypoperfusion (CCH). CCH causes persistent ischemia and hypoxia in brain tissue, activates microglia to trigger a neuroinflammatory cascade, promotes pro-inflammatory factor storm, blood–brain barrier (BBB) destruction and peripheral immune cell infiltration, and eventually is thought to promote neuronal death, white matter lesions, and cognitive domain damage [3]. Recent studies have found [4] that the imbalance of the gut–brain axis may play a candidate upstream role in VCI and that intestinal mucosal barrier damage and gut dysbiosis cause the metabolites produced by the gut microbiota to penetrate the hierarchical biological barriers (comprising both the intestinal epithelial barrier and the blood–brain barrier) and enter the bloodstream, which aggravates central neuroinflammation by activating systemic inflammation and destroying the BBB [5] (In this context, the ‘hierarchical barriers’ refer to the physical, chemical, and immunological defenses that restrict the paracellular and transcellular passage of luminal microbes and their metabolites into the host internal milieu [5,6].). Cerebral ischemia further exacerbates intestinal barrier damage, resulting in the formation of a vicious cycle of “leaky gut–neuroinflammation–cerebral ischemia”. A vicious cycle is formed. It is important to note that VCI encompasses diverse etiological subtypes, including post-stroke cognitive impairment, subcortical ischemic vascular disease, cerebral amyloid angiopathy-related cognitive impairment, and mixed Alzheimer’s/vascular pathology. Chronic cerebral hypoperfusion is most relevant to subcortical ischemic vasculopathy, whereas other subtypes may involve distinct pathomechanisms. Therefore, the gut–barrier–neuroinflammation model proposed in this review should be interpreted as potentially applicable to selected VCI phenotypes rather than the entire clinical spectrum. Although existing drugs (e.g., cholinesterase inhibitors) [5] and non-pharmacological interventions (cognitive training, acupuncture) [7] may partially alleviate the symptoms, there is a lack of specific treatment targeting the core of vascular pathology. Therefore, an in-depth analysis of the mechanism of action of the gut mucosal barrier–neuroinflammation interaction network will provide a novel strategy for early warning and targeted intervention of VCI.

## 2. Methodology

This is a narrative review, not a systematic review. To ensure transparency, we documented our search strategy, selection criteria, screening process, and evidence classification as follows.

### 2.1. Literature Search, Screening, and Data Extraction

We systematically searched PubMed, Web of Science, and CNKI for articles published from January 2015 to May 2026 (final search on 15 May 2026), combining three thematic groups using AND: (a) VCI-related terms (e.g., “vascular cognitive impairment”, “VCI”, “vascular dementia”, “chronic cerebral hypoperfusion”, “CCH”); (b) neuroinflammation-related terms (e.g., “neuroinflammation”, “microglia”, “blood-brain barrier”, “BBB”); and (c) gut–brain axis terms (e.g., “gastrointestinal microbiome”, “gut microbiota”, “gut-brain axis”, “intestinal barrier”, “short-chain fatty acids”, “TLR4”, “lipopolysaccharide”). For CNKI, equivalent Chinese terms were used.

Inclusion criteria: peer-reviewed original or review articles in English or Chinese that addressed mechanisms linking gut barrier function, the gut microbiota, neuroinflammation, and VCI or related conditions (e.g., CCH, CSVD). Exclusion criteria: conference abstracts, case reports, editorials, opinion pieces, studies not directly relevant to the gut–brain axis in VCI, and studies without central nervous system outcomes. Two independent reviewers (J.R.D. and L.S.) screened titles/abstracts and full texts using a standardized form capturing study design, model system, sample size, gut microbiota/metabolite measurements, neuroinflammatory/cognitive outcomes, and key mechanistic findings; disagreements were resolved by consensus (with third-reviewer arbitration if needed). Inter-reviewer agreement was excellent (κ = 0.86 for title/abstract; κ = 0.91 for full-text).

The initial search yielded 847 records (PubMed: 412; Web of Science: 298; CNKI: 137). After removing duplicates (*n* = 143), 704 records were screened; 186 full-text articles were assessed, and 89 were included (43 original research, 46 reviews). Manual reference screening of included articles added 37 pre-2015 studies for mechanistic context, bringing the total to 126 sources.

### 2.2. Evidence Grading, Synthesis of Conflicting Findings, and Use of Primary vs. Review Articles

Recognizing the variable methodological rigor across the gut–brain axis literature in VCI, we applied a four-level evidence classification to each mechanistic claim: Level 1—human clinical evidence (patient cohorts, case–control, or interventional trials); Level 2—animal model evidence using gut-targeted interventions or transgenic models; Level 3—in vitro/cellular mechanistic studies; Level 4—translational/speculative hypotheses not yet tested in VCI (extrapolated from other diseases or indirect evidence). Mechanistic claims in the manuscript are annotated accordingly.

For discordant results (e.g., divergent microbial taxa or context-dependent SCFA effects), we considered findings replicated in at least two independent studies as more robust, systematically noted methodological heterogeneity as potential sources of discordance, reported negative/null findings alongside positive ones, and transparently acknowledged unresolved discrepancies as evidence gaps. Conflicts were resolved through narrative discussion, not meta-analysis. We also distinguished primary studies from review articles, deriving mechanistic claims primarily from primary data; reviews were used for contextualization and gap identification, and any assertion supported only by review articles without primary VCI-relevant data was labeled as Level 4 (speculative).

## 3. Epidemiology, Clinical Features, and Current Therapeutic Strategies for VCI

VCI is a class of cognitive decline syndromes triggered by cerebrovascular lesions and their associated risk factors [8,9]. Its core pathophysiological mechanism lies in the long-term hypoperfusion state of brain tissue, which is associated with activation of a vicious cycle of chronic ischemia and hypoxia. This ongoing crisis of energy metabolism is not a static event but a continuous process of progressive nerve damage and structural and functional destruction of neural networks [10]. Neuronal function is impaired in hypoxic environments, and synaptic plasticity is reduced, ultimately leading to extensive white matter fiber bundle damage and atrophy of key brain regions [11,12]. Notably, the clinical progression of VCI often shows the characteristics of stepwise deterioration, which is closely associated with recurrent cerebrovascular events, each of which can be a turning point for a significant decline in cognitive function [13]. VCI occupies a unique position in the spectrum of cognitive impairment disorders, being the only type with a clear window of intervenability and potential reversibility. Unlike the irreversible pathological protein deposition in neurodegenerative lesions such as AD, the vascular etiology of VCI points to modifiable risk factors (e.g., hypertension, diabetes mellitus, hyperlipidemia) and improved cerebral hemodynamics [14]. Therefore, reducing the risk of developing VCI and delaying its conversion to VD by aggressively controlling vascular risk factors, optimizing cerebral perfusion, and implementing early screening to identify at-risk populations has become one of the most promising core directions in the global dementia prevention and control strategies, the importance of which has been repeatedly emphasized in the authoritative literature [15].

Over the past decade, significant advances have been made in understanding VCI pathophysiology. Landmark studies have established that chronic cerebral hypoperfusion is associated with activation of a cascade of molecular changes, including energy imbalance, glutamate excitotoxicity, acidotoxicity, and oxidative stress, which collectively provoke microglial activation and the release of pro-inflammatory cytokines that recruit systemic immune cells into the central nervous system [16]. The recognition that BBB dysfunction can occur at various stages of chronic cerebral hypoperfusion and that its permeability increases has fundamentally reshaped our understanding of VCI progression [17]. Furthermore, the emerging role of the gut microbiota in modulating neuroinflammation and vascular pathology represents a paradigm shift in VCI research [18]. Recent large-scale cohort studies have begun to reveal the modifiable risk factor spectrum of VCI, providing new targets for early intervention [13]. Advances in imaging technology, particularly the application of high-resolution MRI and PET imaging, have enabled in vivo assessment of BBB integrity and neuroinflammatory status, offering non-invasive tools for early diagnosis and disease monitoring of VCI [17].

VCI encompasses two main categories in clinical practice: one is the classic VD, which is usually triggered by a significant stroke event or extensive small subcortical vascular lesions, and the other involves the complexity of coexisting vascular factors with neurodegenerative pathology such as AD (mixed dementia). Epidemiologic data show [19] that the overall prevalence of mild cognitive impairment in China’s older adults over the age of 65 is 20.8%, which means that more than one-fifth of older adults are at risk for cognitive decline. More seriously, the proportion of mild cognitive impairment caused by cerebrovascular disease and vascular risk factors is as high as 42.0%. With the acceleration and deepening of the process of population aging in China, the disease burden, caregiving pressure, and socioeconomic costs associated with this large base are increasing geometrically, posing a serious public health challenge. Current therapeutic strategies for VCI can be categorized into pharmacological and non-pharmacological approaches. Pharmacological treatments include cholinesterase inhibitors (e.g., donepezil), which provide symptomatic cognitive benefit but do not directly target underlying vascular pathology. Non-pharmacological interventions—including cognitive rehabilitation, transcranial magnetic stimulation, and acupuncture—have shown variable efficacy in improving cognitive function. Notably, emerging evidence suggests that some of these interventions, particularly acupuncture and cognitive training, may exert beneficial effects partly through modulation of the gut–brain axis, though this remains largely speculative and requires mechanistic validation. Traditional Chinese Medicine approaches, which emphasize holistic regulation of multiple systems, have been investigated as potential modulators of gut microbiota composition and inflammatory status in VCI models. However, the precise mechanisms linking these therapeutic modalities to the gut mucosal barrier–neuroinflammation network remain poorly defined, and rigorous clinical trials are needed to establish causality. Pharmacologic treatments include cholinesterase inhibitors (e.g., donepezil hydrochloride), which are effective but do not directly target the core pathologic mechanisms of VCI [6,20]. Non-pharmacological treatments include cognitive rehabilitation training, which improves cognitive function through specific training tasks [21]; transcranial magnetic stimulation and transcranial electrical stimulation, which improve cognitive function by modulating the neural activity of the cerebral cortex [22]; and acupuncture treatment, as one of the traditional medical techniques of traditional Chinese medicine (TCM), which is notable in the treatment of VCI. According to TCM, the occurrence of VCI is related to the imbalance of yin and yang, and acupuncture on some brain-related acupoints, such as Baihui [23], Governor Vessel, Bladder Meridian, etc., can wake up the brain and open the orifices, improve the excitability of nerves, and enhance cognitive ability [24]; it also contains psychotherapy, which can alleviate the stress and anxiety of some patients’ lives and reduce the depressive symptoms [25]. Despite the growing wealth of prevention and treatment tools, the complexity of VCI dictates that its management remains a great challenge. Its etiology is highly heterogeneous (large vessel lesions, small vessel disease, hypoperfusion, etc.), which together lead to impaired blood supply to brain tissue, triggering chronic neuronal hypoxia, energy failure, and even irreversible necrosis of neurons. No drugs specifically targeting the gut–brain axis are currently approved for VCI. Cholinesterase inhibitors are symptomatic; non-pharmacological interventions show some efficacy, but their precise mechanisms linked to the gut barrier–neuroinflammation network remain unverified and largely speculative [26,27]. Therefore, the direction of future breakthroughs lies in deepening the understanding of the multi-level pathological mechanism of VCI, promoting the research and development of vascular-targeted therapeutic drugs, and optimizing individualized, multimodal, comprehensive non-pharmacological interventions, with the aim of achieving an effective interception of cognitive function before it declines significantly and ultimately alleviating the heavy burden it imposes.

Beyond the traditional binary classification, VCI is now recognized as a highly heterogeneous clinical entity encompassing multiple etiological subtypes. These include (a) post-stroke cognitive impairment (PSCI) resulting from single strategic infarcts or multiple territorial infarcts; (b) subcortical ischemic vascular disease characterized by extensive white matter hyperintensities (WMH) and lacunar infarcts; (c) cerebral amyloid angiopathy-related cognitive impairment; and (d) mixed AD/vascular pathology. WMH, a key MRI marker of CSVD, is increasingly recognized as arising from chronic hypoperfusion, endothelial dysfunction, BBB disruption, and extravasation of inflammatory mediators. Importantly, neuroinflammation and amyloid deposition may independently contribute to WMH progression, suggesting that the gut barrier–neuroinflammation model may be most relevant to certain VCI subtypes (particularly subcortical ischemic vasculopathy) while being less applicable to others such as strategic infarction. This heterogeneity must be considered when interpreting gut microbiome and inflammatory biomarker findings, as microbial signatures and inflammatory profiles may differ substantially across VCI phenotypes.

The clinical classification of VCI has been refined by consensus criteria including the AHA/ASA and DSM-V diagnostic frameworks, which help distinguish cases with predominantly vascular pathology from those with mixed pathology. Neuroimaging-based subtyping—such as the atrophy-driven classification into Frontal, Subcortical/Temporal, and Parietal subtypes—further provides evidence in animal models that VCI is not a unitary disease but a spectrum of conditions with distinct pathological drivers and clinical trajectories.

## 4. Neuroinflammation and Chronic Cerebral Ischemia as Major Drivers of VCI Pathogenesis

VCI is not a simple lesion driven by a single etiologic factor but a dynamic network formed by the complex interaction of multi-system and multi-level pathological factors. In this intricate network, chronic cerebral ischemia–hypoxia and the ensuing storm of neuroinflammation constitute the driving force behind the progressive decline in cognitive function. They reinforce and amplify each other, forming a vicious cycle that is difficult to break, ultimately leading to structural damage and functional decompensation of the neural network [28,29].

### 4.1. Cerebral Small Vessel Disease (CSVD)—The Pathologic Basis of VCI and the Initiating Platform of Neuroinflammation

CSVD is the main pathologic basis of VCI, involving small arteries, venules, and capillaries in the brain, as well as the sophisticated structures they constitute—the neurovascular unit [30]. The neurovascular unit is a functional complex of neurons, astrocyte endfeet, vascular endothelial cells, pericytes, basement membranes, and extracellular matrix working in concert to maintain the integrity of the BBB and to accurately regulate cerebral blood flow and exchange of substances [31]. Standardized visual rating scales for CSVD imaging include the Fazekas scale (0–3 for deep WMH and 0–3 for periventricular WMH, total 0–12), medial temporal atrophy (MTA) score (0–4), and counts of microbleeds, lacunes, and perivascular spaces (PVS). The BIOCIS cohort, using this system, found that Fazekas and MTA scores significantly correlate with cognitive performance even after adjusting for age and education [32,33,34].

#### 4.1.1. Vascular Injury Driven by Risk Factors

Risk factors such as aging, hypertension, and diabetes mellitus are major drivers of CSVD. They cause structural and functional abnormalities in small cerebral blood vessels (e.g., lumen stenosis/occlusion, vessel rupture, blood–brain barrier leakage, neuroinflammation, etc.), which ultimately lead to cognitive impairment [10].

#### 4.1.2. BBB Disruption—A Trigger of Neuroinflammation

The key events in the progression of CSVD are progressive damage and leakage of the BBB. A healthy BBB severely restricts the free access of substances from the blood, including immune cells and inflammatory factors, into the brain parenchyma. In CSVD, down-regulated or structurally disordered expression of tight junction proteins between endothelial cells, loss of pericytes, and dysfunction of astrocyte end-feet collectively lead to abnormally increased BBB permeability [35], allowing plasma proteins (e.g., fibrinogen), immune cells (e.g., monocytes, neutrophils), and pro-inflammatory factors in the peripheral circulation to infiltrate into the brain parenchyma and to activate pro-inflammatory factors that reside in the CNS microglia directly [29,36]. Beyond tight junction disruption, altered expression of transporters represents another important hallmark of BBB dysfunction in VCI. The BBB expresses a variety of transporters—including efflux transporters such as P-glycoprotein (P-gp/ABCB1) and breast cancer resistance protein (BCRP/ABCG2), as well as solute carrier (SLC) family transporters—that regulate the bidirectional transport of nutrients, metabolites, and exogenous substances [28,29]. Dysregulation of these transporters in chronic cerebral hypoperfusion may significantly influence the transport of microbiota-derived metabolites and other circulating factors across the BBB [15]. For instance, reduced P-gp function could allow for increased brain accumulation of potentially neurotoxic microbial metabolites, while altered SLC transporter expression might affect the uptake of beneficial SCFAs into the brain parenchyma [24]. The BBB contains transport systems specifically optimized for microbial metabolite processing: monocarboxylate transporters MCT1/MCT2 mediate SCFA transport, while efflux systems including P-glycoprotein clear harmful bacterial metabolites [37].

#### 4.1.3. Cascade Amplification and Dynamic Evolution of Neuroinflammation

Under the stimulation of chronic ischemia and hypoxia and leakage of substances, the neuroinflammatory response of brain tissues is ignited and shows a dynamic, stage-by-stage cascade amplification [38]. Neuroinflammation after cerebral ischemia shows a dynamic evolution: ① Acute phase: Resting microglia are rapidly activated and transformed into phagocytosis (M1-type predominantly) [39], which migrate to the site of injury and phagocytose to remove necrotic cellular debris and leaked plasma proteins [40]. At the same time, small amounts of pro-inflammatory factors and reactive oxygen species are released to initiate inflammatory signaling. ② Subacute phase: The inflammatory response enters the stage of outbreak and diffusion. Continuous hypoxic stimulation, massive production of reactive oxygen species, and feedback from the initial pro-inflammatory signals drive microglia and infiltrating macrophages to secrete large amounts of potent pro-inflammatory cytokines, chemokines, and adhesion molecules. Chemokines recruit more peripheral immune cells across the damaged BBB into the brain tissue, creating a vicious cycle. Reactive oxygen species and inflammatory mediators work together to further damage adjacent neurons, oligodendrocytes, and vascular endothelial cells. ③ Chronic phase: Inflammation enters a state of sustained low-grade combustion, which is the most harmful phase in the progression of VCI. Continued stress from inflammatory mediators and oxidative stress is thought to promote mitochondrial dysfunction, oxidative DNA damage, and endoplasmic reticulum stress. These injuries ultimately trigger ongoing programs such as apoptosis and ferroptosis, resulting in continued loss of neurons, oligodendrocytes, and vascular cells [41]. ④ In the recovery period: In the case of attenuated injury stimuli or effective intervention, the expression of anti-inflammatory factors increases, and some microglia are transformed into the M2 type with anti-inflammatory and repair functions, secreting neurotrophic and pro-repair factors in an attempt to remove inflammatory mediators and promote neovascularization and synaptic remodeling. The transition from the subacute to the chronic phase—and whether inflammation ultimately resolves—is critically modulated by the dynamic equilibrium between M1 and M2 microglial phenotypes, the efficiency of damaged cellular debris clearance, and the persistence of peripheral immune cell infiltration [10]. In the context of recurrent ischemic insults or sustained systemic endotoxemia (as discussed in Section 4), this equilibrium is pathologically skewed towards the chronic, low-grade inflammatory state, which is the principal driver of progressive neuronal loss and white matter rarefaction in VCI [10,28].

### 4.2. Hypoxia: A Driver of Neuroinflammation and a Direct Destroyer of the Neurovascular Unit

Hypoxia is not only a direct consequence of CSVD and chronic cerebral hypoperfusio but is also a central driving force in initiating and exacerbating neuroinflammation and destroying the neurovascular unit.

#### 4.2.1. Direct Neuronal Damage

Hypoxia is thought to promote impaired mitochondrial oxidative phosphorylation, drastically reduced adenosine triphosphate (ATP) production, and an energy crisis that is associated with activation of ion pump dysfunction, leading to neuronal depolarization, excitotoxicity, and ultimately calcium overload and acute neuronal death.

#### 4.2.2. Glial Cell Activation and Inflammatory Factor Storm

Hypoxia is one of the strongest signals to activate microglia and astrocytes. Hypoxia-inducible factor-1α is stably expressed in hypoxic environments and acts as a key transcription factor to directly regulate the expression of a large number of pro-inflammatory genes. Activated glial cells release pro-inflammatory cytokines, chemokines, and reactive oxygen species in large quantities, forming a “storm of inflammatory factors” that attacks neurons and vascular structures [42].

#### 4.2.3. Defective Autophagy—An Amplifier of Inflammation

Autophagy is an important mechanism for cells to remove damaged organelles and proteins and maintain homeostasis. Studies have shown that hypoxia-induced autophagy defects can activate the inflammatory response of microglia [43]. Hypoxia induces autophagy initiation by inhibiting the mechanistic target of rapamycin complex 1 (mTORC1) signaling pathway on the one hand and impairs the fusion of autophagosomes with lysosomes and lysosomal function on the other hand, leading to a large accumulation of autophagosomes, activation of inflammatory signals, and sustained stimulation of pro-inflammatory responses in microglia. Beyond the mTORC1 pathway, the AMP-activated protein kinase (AMPK) pathway, as a key autophagy regulator that senses energy stress, can induce autophagy independently of mTORC1 under hypoxic conditions [42]. Furthermore, the Beclin-1/VPS34 complex, which is essential for autophagosome nucleation, has been shown to be dysregulated in chronic cerebral ischemia, contributing to impaired autophagic flux [41]. The accumulation of autophagosomes due to impaired lysosomal fusion not only activates inflammatory signaling but also promotes neuronal apoptosis through the activation of caspase-dependent pathways [43]. Studies have found that in CCH models, autophagy plays an important role in cognitive dysfunction: URB597 improves CCH-induced cognitive impairment by inhibiting mTOR-dependent autophagy [44]; resveratrol activates autophagy through the AKT/mTOR signaling pathway to improve cognitive function in CCH rats [45].

#### 4.2.4. A Vicious Cycle of BBB Destruction

Hypoxia directly damages vascular endothelial cells, decreasing their expression of tight junction proteins and increasing permeability. Activation of microglia increases BBB permeability by destroying BBB structural integrity and endothelial cell dysfunction, allowing immune cells and potentially harmful substances to enter the brain, exacerbating inflammation, brain cell damage, and apoptosis [46], and forming a vicious cycle of “hypoxia → inflammation → BBB destruction → more inflammatory factors/cellular infiltration → more severe hypoxia/inflammation” vicious cycle.

### 4.3. Chronic Cerebral Hypoperfusion as a Major Upstream Driver

CCH is a major upstream driving force in a substantial subset of VCI patients, particularly those with subcortical ischemic vascular disease. The root cause of CCH is the chronic and persistent reduction in cerebral blood flow (CBF), which cannot meet the normal metabolic demand of brain tissue. Abnormal operation of cerebral blood vessels can lead to vascular structural lesions, altered cerebral hemodynamics, reduced cardiac output, and altered blood composition, which can disrupt the regulation of cerebral blood flow leading to CCH, which in turn can trigger ischemic neuronal damage and cognitive dysfunction. Long-term CCH causes cerebral tissue ischemia and hypoxia, which in turn is associated with activation of a series of pathological changes, including neuroinflammation, oxidative stress, BBB destruction, and white matter lesions. These pathological changes interact with each other and eventually lead to cognitive dysfunction [47]. Studies have shown that CCH induces microglia activation, promotes the release of a variety of pro-inflammatory cytokines, and initiates an inflammatory response, which in turn is thought to promote neuronal damage and cognitive dysfunction [48].

### 4.4. Other Factors

The pathogenesis of VCI is also associated with genetic factors, cerebrovascular amyloidosis, iron metabolism disorders, and many other factors [49]. For example, autosomal dominant cerebral arteriopathy with subcortical infarcts and leukoencephalopathy (CADASIL) is one of the inherited small vessel diseases that eventually transforms into cognitive impairment. In addition, Amyloid Precursor Protein (APP) variation or duplications in cerebral amyloid angiopathy lead to overproduction and deposition of amyloid β-protein (Aβ) in the walls of leptomeningeal vessels, which promotes the occurrence of VCIs [50]. Aβ activates signaling pathways on the surface of microglial cells, such as nuclear factor-κB (NF-κB) and extracellular signal-regulated kinase/mitogen-activated protein kinase (ERK/MAPK), and induces pro-inflammatory gene expression and cytokine/chemokine production [51].

**Figure 1:**
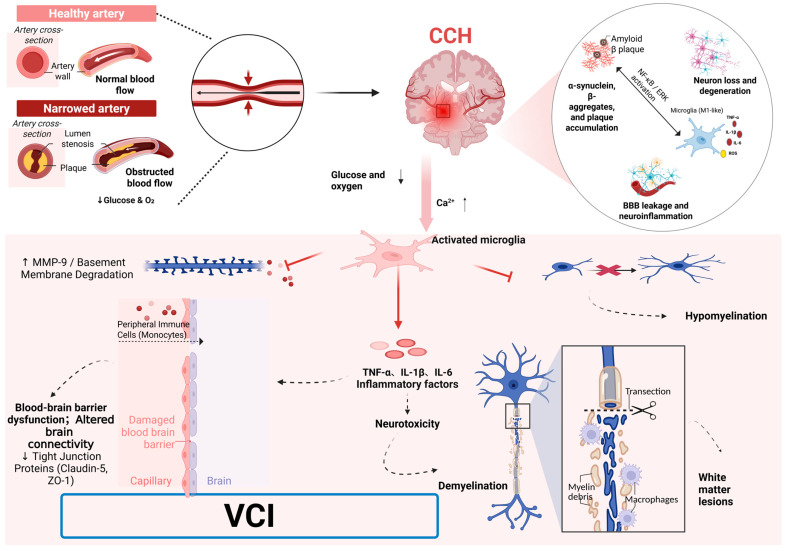
Core pathogenesis of VCI: deep intertwining and cascade amplification of neuroinflammation and chronic cerebral ischemia. Red indicates pathological states; blue indicates normal structures. Solid/dashed arrows indicate direct/indirect actions. ↑/↓ represent upregulation/downregulation; ⊥ indicates inhibition; ✂ indicates physical transection.

It can be seen that the core pathogenesis of VCI is a chain reaction process with CCH as the initiating point, which is associated with activation of persistent cerebral tissue ischemia and hypoxia and then activates neuroinflammation (microglia/astrocyte activation, pro-inflammatory factor storm, and peripheral immune cell infiltration). Neuroinflammation and ischemia–hypoxia are mutually driven and synergistically amplified, which together lead to neurovascular unit dysfunction (especially BBB destruction), neuronal and oligodendrocyte death, and disruption of neural network connectivity. Other factors such as genetic factors, cerebrovascular amyloidosis, and others, either through direct damage to the vasculature or by exacerbating inflammation/oxidative stress, are deeply involved in and complicate this core pathologic network (Figure 1). A deeper understanding of these mechanisms, particularly neuroinflammation and vasculopathy, is important for the future development of novel therapeutic strategies targeting blockade of the inflammatory cascade, improvement of cerebral perfusion, and protection of the neurovascular unit in order to curb VCI, a major threat to cognitive health in the elderly.

The left panel illustrates the vascular pathology of VCI: a healthy artery (top) with a normal lumen and blood flow versus a narrowed artery (bottom) with plaque formation leading to obstructed blood flow, resulting in reduced glucose and oxygen delivery to brain tissue. The center panel depicts the central pathogenic cascade: chronic cerebral hypoperfusion (CCH) activates microglia, which release inflammatory factors and promote amyloid β plaque accumulation. This is associated with activation of neuroinflammation, blood–brain barrier (BBB) leakage, reactive astrogliosis, and altered brain connectivity. The right panel shows the downstream consequences: BBB dysfunction with capillary damage allows inflammatory factors and neurotoxic substances to enter the brain parenchyma, leading to demyelination (with myelin debris and macrophage infiltration), white matter lesions, and ultimately VCI. Arrows indicate the feedforward amplification of neuroinflammation and the vicious cycle between BBB disruption and inflammatory injury. CCH: chronic cerebral hypoperfusion; BBB: blood–brain barrier; VCI: vascular cognitive impairment. This scheme illustrates the core pathogenesis for CCH-driven subtypes; other VCI subtypes may involve additional mechanisms.

## 5. Gut–Brain Axis Imbalance: The Core Hub of the Interaction Between Intestinal Mucosal Barrier Destruction and Neuroinflammation in VCI

As a complex two-way communication network connecting the intestinal microenvironment and the central nervous system, dysfunction of the gut–brain axis has been recognized as a candidate upstream contributor and important factor driving the occurrence and development of VCI. In this axis, the integrity of the intestinal mucosal barrier and the homeostasis of the intestinal flora play important roles, which, together with neuroinflammation, BBB destruction, and chronic cerebral ischemia, form a pathological cycle and together constitute an important contributing factor in the pathophysiological network of VCI [16]. It is important to note that the evidence supporting the gut–brain axis hypothesis in VCI comes from heterogeneous sources with substantially different levels of certainty. To provide a transparent framework for evaluating the strength of the evidence, we adopt the following classification throughout this section: (1) human clinical evidence—findings from patient cohorts, case–control studies, and clinical trials; (2) animal model evidence—data from experimental CCH models (e.g., bilateral common carotid artery occlusion, BCCAo); (3) in vitro/cellular evidence—mechanistic studies using cell culture systems; (4) translational/speculative hypotheses—proposed mechanisms that have not yet been directly tested in humans or relevant animal models.

### 5.1. Intestinal Mucosal Barrier Damage: An Upstream Event Driving Neuroinflammation

The intestinal mucosal barrier is one of the largest and most critical biological interfaces in the body, comprising a combination of physical, chemical, immune, and biological barriers. Its core function is to selectively allow for nutrient absorption while tightly blocking large numbers of microorganisms, toxins, and harmful antigens in the gut from entering the circulation [52].

#### 5.1.1. The Starting Point of the “Leaky Gut” Pathological Cycle

Under the effects of aging, chronic stress, high-fat and high-sugar diets, and antibiotic abuse, the intestinal mucosal barrier suffers sustained damage, and the structural disintegration and dysfunction of the intestinal epithelial intercellular tight junctions occur. Down-regulation of the expression, mislocalization, or altered phosphorylation status of key Tight Junction (TJ) proteins is thought to promote abnormally high paracellular permeability. At the same time, the barrier defense is further weakened by a thinning of the mucus layer, a decrease in antimicrobial peptides secreted by PAN cells, and a decrease in intestinal secretory immunoglobulin A (sIgA) levels. This state of total breakdown of intestinal barrier function is known as “leaky gut” [L4: Translational hypothesis; direct evidence in VCI is limited] [53].

#### 5.1.2. Microbiota Translocation and Systemic Inflammatory Outbreaks

The consequence of “leaky gut” is the translocation of intestinal flora and its metabolites/structural components (e.g., lipopolysaccharides, peptidoglycan, and flagellin in Gram-negative bacteria; lipophosphatidic acid in Gram-positive bacteria) as well as incompletely digested antigenic macromolecules of food to the portal circulation and to the somatic circulation [L2: Animal models; L1: Human correlational in other diseases] [54]. Pathogen-associated molecular patterns such as lipopolysaccharides (LPS) are recognized and bound by pattern recognition receptors on circulating immune cells and vascular endothelial cells, particularly Toll-like receptor 4 (TLR4).

#### 5.1.3. TLR4 Activates Systemic and Central Inflammatory Responses

Translocated microbial products, particularly LPS from Gram-negative bacteria, activate Toll-like receptor 4 (TLR4) on circulating immune cells and cerebral vascular endothelial cells, inducing endothelial dysfunction, adhesion molecule upregulation, and BBB tight junction disruption, thereby facilitating peripheral immune cell infiltration into the brain parenchyma [31,46]. Inaba et al. [55] demonstrated in a high-fat diet (HFD)-induced obese mouse model with chronic cerebral hypoperfusion that HFD feeding caused gut dysbiosis and increased intestinal permeability, resulting in elevated plasma LPS and pro-inflammatory cytokines, with higher LPS levels and stronger neuroinflammation in white matter lesions. Critically, in TLR4-knockout mice, despite similar obesity and gut dysbiosis, HFD did not exacerbate post-ischemic cognitive impairment or white matter lesion severity, confirming that LPS-TLR4 signaling mediates obesity-related exacerbation of cognitive impairment and white matter lesions after cerebral ischemia [L2: Causal evidence from TLR4-KO mice] [55]. Similarly, LPS-induced TLR4/NF-κB pathway activation in aged mice is involved in microbiota–gut–brain axis-related neuroinflammation and cognitive decline [56]. Beyond LPS-TLR4 signaling, clinical studies have identified plasma matrix metalloproteinase-9 (MMP-9), lipoprotein-associated phospholipase A2 (Lp-PLA2), interleukin-6 (IL-6), and tumor necrosis factor-alpha (TNF-α) as independent correlates of VCI severity in CSVD patients, with levels positively correlated with cognitive impairment severity [L1: Human cross-sectional cohort, n = 400] [57,58]. Decreased expression of the immunoregulatory molecule V-domain immunoglobulin suppressor of T cell activation (VISTA) has also been associated with elevated pro-inflammatory cytokines and increased VCI risk in patients with cardiovascular risk factors. Collectively, these findings indicate that neuroinflammation in VCI is driven by a complex network of inflammatory mediators, with the gut-barrier axis representing one—but not the only—important source of systemic inflammation.

#### 5.1.4. Bidirectional Reinforcement of Cerebral Ischemia and Intestinal Barrier

The reciprocity of this interaction is mediated by neuroendocrine activation: cerebral ischemia/hypoxia overactivates the hypothalamic-pituitary-adrenal (HPA) axis and sympathetic nervous system, releasing corticotropin-releasing hormone (CRH) and catecholamines, which impair mucin (MUC2) and antimicrobial peptide expression, thereby increasing intestinal paracellular permeability [L2: Animal models; neuroendocrine pathway established in stroke, not specifically VCI] [59]. In a BCCAo rat model of CCH, Su et al. demonstrated that fecal microbiota transplantation (FMT) and SCFA supplementation reversed CCH-induced decreases in fecal and hippocampal acetate/propionate, inhibited microglial and astrocytic activation, promoted M1 → M2 microglial polarization, reduced neuronal and microglia-mediated synaptic loss, maintained synaptic vesicle fusion/release, and ultimately ameliorated cognitive impairment [60]. FMT and SCFAs treatment also reduced neuronal loss and microglia-mediated synaptic loss, maintaining the normal process of synaptic vesicle fusion and release, thereby improving synaptic plasticity. These effects ultimately inhibited CCH-induced cognitive impairment [L2: Animal intervention model, FMT and SCFA supplementation] [60]. These findings support a bidirectional interaction and suggest a self-perpetuating cycle, although direct evidence in human VCI remains lacking [59,60].

### 5.2. The Interaction Between the Gut Microbiota and the Immune System

The gut microbiota is an extremely complex ecosystem, and its composition and functional homeostasis are essential for maintaining host health. Conversely, its imbalance is a central factor driving a variety of chronic diseases, including VCI. A continuous, dynamic dialog between the gut flora and the host immune system, particularly the intestinal mucosal immune system, is key to modulating intestinal barrier function and systemic/central inflammatory states. A healthy gut microbiota produces beneficial metabolites such as short-chain fatty acids (SCFAs). SCFAs may regulate brain function and immunity via the BBB [61] [L2: Predominant; L4 for human translation]. The neuroprotective potential of SCFAs is largely derived from preclinical studies, with large-scale population trials still lacking [56]. Notably, the effects of SCFAs on neuroinflammation are dose-dependent and context-dependent. In pathological contexts such as α-synuclein aggregation, SCFAs may paradoxically drive microglial activation and exacerbate neural damage [L2/L4: Extrapolated from Parkinson’s models; not yet tested in VCI]. Simply supplementing specific metabolites without considering the overall microbial ecology may be counterproductive [62]. The intestinal mucosal immune system (including antimicrobial peptides secreted by intestinal epithelial cells, secretory immunoglobulin A, and intrinsic and adaptive immune cells) is a key barrier to maintaining intestinal homeostasis and defense against pathogens and is directly regulated by flora [63,64]. Dysregulated gut flora is thought to be an important source of neuroinflammation, although this evidence derives primarily from Parkinson’s disease models and requires verification in VCI [L4: Extrapolated from other diseases] [65]. For example, dysbiosis activates pathways such as NOD-like receptor family pyrin domain containing 3 (NLRP3) inflammatory vesicles, which promotes cytokine release, increases intestinal permeability, and disrupts the BBB; at the same time, these signals activate CNS microglial cells, which alter brain structure and function [L2: Animal models; L4 for direct VCI evidence] [66]. Intestinal epithelial cells form a physicochemical barrier separating flora from host immunity [67]. Intestinal glial cells undergo “reactive gliosis” in response to inflammatory stimuli, releasing pro-inflammatory cytokines and other signaling molecules, which are involved in bidirectional inflammatory regulation in the gut–brain [68].

**Figure 2:**
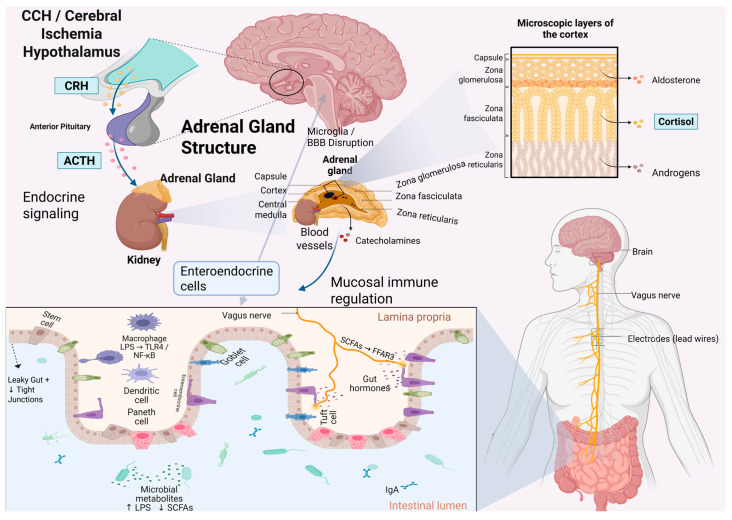
Imbalance of the gut–brain axis: the core hub of the interaction between intestinal mucosal barrier disruption and neuroinflammation in VCI. Blue indicates endocrine/HPA axis signaling; yellow indicates adrenal gland structures; pink indicates mucosal immunity and neural pathways. Solid/dashed arrows indicate direct/indirect regulatory actions. ↑/↓ represent upregulation/downregulation.

The centrality of the gut–brain axis in the pathomechanism of VCI is becoming increasingly prominent. Disruption of the intestinal mucosal barrier is thought to promote translocation of intestinal flora products (e.g., lipopolysaccharides), triggering systemic inflammation, disruption of the BBB, recruitment of peripheral immune cells for infiltration, activation of microglia, and outbreaks of CNS neuroinflammation. Neuroinflammation and cerebral ischemia, in turn, feedback damage the intestinal barrier, forming a vicious cycle. At the same time, intestinal microecological dysbiosis is thought to promote a decrease in protective SCFAs, an increase in harmful metabolites, and overactivation of inflammatory vesicles, which further is associated with local and systemic inflammation and affects the central nervous system through multiple pathways (Figure 2). Understanding this complex network of interactions opens up a new perspective for the prevention and treatment of VCI: targeting the repair of the intestinal mucosal barrier, regulation of intestinal flora, inhibition of key inflammatory pathways, and supplementation of beneficial metabolites are expected to be effective means of preventing and treating VCI in the future by disrupting the “gut–brain inflammatory axis.”

The left panel depicts the healthy gut–brain axis: The intact intestinal mucosal barrier (with tight junctions, mucus layer, and antimicrobial peptides) maintains gut homeostasis; beneficial gut microbiota produce SCFAs that cross the BBB and support normal brain function. The center panel shows the “leaky gut” pathological state: disruption of tight junction proteins allows for the translocation of LPS and other microbial products into the portal and systemic circulation. The right panel illustrates the central neuroinflammatory consequences: circulating LPS activates TLR4 on cerebral vascular endothelial cells and peripheral immune cells, triggering systemic inflammation, BBB disruption, and microglial activation. Pro-inflammatory cytokines (TNF-α, IL-1β, IL-6) and infiltrating immune cells amplify CNS neuroinflammation, leading to neuronal damage and cognitive impairment. Arrows indicate the bidirectional reinforcement: cerebral ischemia/hypoxia feeds back to further damage the intestinal barrier via neuroendocrine pathways (HPA axis and sympathetic activation), completing the vicious cycle. The proposed vicious cycle is supported primarily by preclinical evidence; human validation is pending.

### 5.3. Gut Microbiota Dysbiosis and Microbial Metabolites in VCI

Accumulating evidence indicates that VCI is associated with significant alterations in gut microbiota composition and microbial metabolite profiles. A systematic review of relevant studies summarized that VCI patients exhibit significant changes in both the structure and abundance of gut microbiota, with candidate taxa identified across multiple independent cohorts [L2/L4, derived from original studies including Liu et al.] [69]. Specifically, Enterobacteriaceae showed modest discriminatory ability (AUC 0.629), indicating weak to moderate separation between groups; this does not support clinical diagnostic application. Other microbial signatures, including Bifidobacterium, Lactobacillus gasseri, and Anaerostipes hadrus, reached AUCs of 0.750–0.792, which warrant further validation but remain exploratory. These findings should be interpreted as candidate signatures requiring prospective validation rather than established diagnostic biomarkers [L1: Human cross-sectional; exploratory candidate signatures] [18]. Table 1 summarizes the major microbial taxa and metabolite changes associated with cerebral ischemia and VCI, along with their proposed biological effects. Among the most extensively studied metabolites is trimethylamine N-oxide (TMAO), generated by gut microbial metabolism of dietary choline. A cross-sectional study demonstrated that plasma choline and TMAO levels were negatively correlated with cognitive scores in CCH patients, and a choline-rich diet exacerbated CCH-induced cognitive impairment by promoting the proliferation of choline-metabolizing bacteria and subsequent TMAO generation [L1: Human cross-sectional, *n* not specified; causality not established] [70]. Short-chain fatty acids (SCFAs), including acetate, propionate, and butyrate, represent another critical class of protective metabolites. In a rat CCH model, FMT and SCFA replenishment reversed decreased fecal and hippocampal acetate/propionate levels, inhibited glial activation, and promoted M1 → M2 microglial polarization, thereby exerting anti-neuroinflammatory effects [L2: Su et al. BCCAo rat model] [60]. Additionally, acetic acid supplementation ameliorated cognitive impairment in chronic cerebral ischemia mice by reducing tau hyperphosphorylation through the FFAR3/Erk signaling pathway [L2: Mouse model of chronic cerebral ischemia] [71].

**Table 1:** Gut microbial taxa and metabolites associated with CCH/VCI and their proposed biological effects.

Microbial Taxa/Metabolite	Alteration in CCH/VCI (Sample Source)	Proposed Biological Effects & Targets	Key Evidence
*Enterobacteriaceae*	Increased Fecal abundance	Positively correlated with peripheral LPS levels and cognitive decline; potential TLR4/NF-κB pathway activation.	Human cohort study [72] (L1)
*Bifidobacterium*	Altered fecal abundance	Associated with cognitive function; may modulate inflammatory responses.	Human cohort study [72] (L1)
*Lactobacillus gasseri*	Altered fecal abundance	May help differentiate PSCI from stroke survivors without cognitive deficits.	Human cohort study [72] (L1)
*Anaerostipes hadrus*	Altered fecal abundance	May help differentiate PSCI from stroke survivors without cognitive deficits.	Human cohort study [72] (L1)
*Verrucomicrobiae*/*Akkermansiaceae*	Decreased Fecal abundance	Promote microglial M2 polarization; inhibit HDAC; activates FFAR2/3.	FMT study [60] (L2)
Trimethylamine N-oxide (TMAO)	Increased Plasma levels	Negatively correlated with cognitive scores; may promote neuroinflammation.	Review [73]; Clinical study [74] (L1)
Short-chain fatty acids (acetate, propionate)	Decreased Fecal and/or hippocampal levels	Inhibits glial activation; promotes M1 → M2 microglial polarization; inhibits tau phosphorylation via FFAR3/Erk pathway.	Animal models [60,71] (L2)

Note: All microbial taxa alterations refer to relative abundance in fecal samples, not circulating plasma levels. Evidence levels: L1 = human clinical; L2 = animal model.

At the methodological level, the Singapore BIOCIS cohort provides an important reference, employing multi-timepoint sampling with dietary control (7-day food diaries and food frequency questionnaires) to improve temporal inference, though definitive causal attribution requires interventional designs integrating metagenomics, metabolomics, blood biomarkers, neuroimaging, and cognitive data [L1: Human prospective cohort; improves temporal inference but does not establish causality] [34]. Table 2 summarizes the major findings from key studies investigating the gut barrier–neuroinflammation axis in VCI across different VCI phenotypes.

Several discordant findings merit discussion. Although SCFAs are predominantly neuroprotective in CCH models, studies in other neurodegenerative contexts have reported pro-inflammatory effects under conditions of α-synuclein aggregation, reflecting context-dependent effects that may vary by disease model, concentration, or administration route—underscoring that simple metabolite supplementation without considering overall microbial ecology may be counterproductive. Additionally, the directionality of changes in specific taxa (e.g., Bifidobacterium) varies across cohorts, likely due to differences in dietary backgrounds, VCI subtypes, and sequencing methodologies. These unresolved discrepancies highlight the need for standardized multi-cohort analyses [75].

**Table 2:** Summary of key evidence for gut barrier–neuroinflammation mechanisms in VCI.

Evidence Category	Model/System	Key Finding	VCI Phenotype	Citation
Gut microbiota composition	Human cohort	Ruminococcus gnavus abundance negatively correlated with MoCA scores; mediated by CBF in bilateral hypothalamus and left amygdala	CSVD-related VCI	Liu et al., 2025 [71]
Microbial metabolites	Human + animal	Plasma choline and TMAO elevated in CCH patients; choline-rich diet promotes TMAO production and exacerbates cognitive impairment	CCH-induced VCI	Li et al., 2025 [74]
SCFAs	Rat BCCAo model	Fecal and hippocampal acetate/propionate decreased; FMT and SCFA supplementation reversed changes, inhibited glial activation, promoted M1→M2 microglial polarization	CCH model	Su et al., 2023 [60]
LPS-TLR4 signaling	Mouse HFD + CCH + TLR4-KO	Plasma LPS elevated; TLR4-KO blocked HFD-induced exacerbation of post-ischemic cognitive impairment and WML severity	Obesity + CCH model	Inaba et al., 2023 [55]
LPS-TLR4/NF-κB	Aged mouse model	LPS-induced TLR4/NF-κB activation mediates neuroinflammation and cognitive decline associated with microbiota–gut–brain axis dysfunction	Aging-related	Wu et al., 2021 [56]
Inflammatory biomarkers	Human CSVD cohort (*n* = 400)	Plasma MMP-9, Lp-PLA2, IL-6, TNF-α are independent risk factors for VCI; levels correlate with cognitive impairment severity	CSVD	Xia et al., 2026 [76]
Intestinal permeability	Human CSVD-CI	Gut microbiota imbalance and increased intestinal permeability associated with cognitive decline	CSVD with CI	Chen et al., 2026 [75]
Gut microbiota signature	Human PSCI	Enterobacteriaceae (AUC = 0.629, indicating weak discrimination), Bifidobacterium, Lactobacillus gasseri, and Anaerostipes hadrus (AUC 0.750–0.792) show modest ability to differentiate PSCI from stroke survivors without cognitive deficits; these are candidate signatures requiring prospective validation	Post-stroke CI	Liu et al., 2025 [71]

Abbreviations: MoCA, Montreal Cognitive Assessment; CBF, cerebral blood flow; TMAO, trimethylamine N-oxide; SCFAs, short-chain fatty acids; FMT, fecal microbiota transplantation; HFD, high-fat diet; TLR4, Toll-like receptor 4; WML, white matter lesion; CSVD, cerebral small vessel disease; PSCI, post-stroke cognitive impairment. Cross-sectional associations identified as ‘independent risk factors’ in the cited study should be interpreted as statistically independent correlates in that specific cohort, not as established causal risk factors, pending prospective validation.

### 5.4. The Dynamic Interplay Between the Gut Microbiota and the Neuroimmune System

The interaction between the gut microbiota and the host immune system is not a static one-way relationship but a continuous, dynamic dialogue that shapes both intestinal homeostasis and central neuroinflammatory states. This bidirectional communication operates through three principal mechanistic pathways:

#### 5.4.1. Neural Pathways

The vagus nerve serves as a direct anatomical link between the gut and the brain, enabling gut-derived signals—including microbial metabolites and inflammatory cytokines—to modulate central neural circuits without crossing the BBB. Afferent vagal fibers express receptors for microbial metabolites such as SCFAs and can transmit inflammatory signals to the nucleus tractus solitarius, which in turn modulates hypothalamic–pituitary–adrenal (HPA) axis activity [L2/L4: Established in general gut–brain axis, not specifically VCI].

#### 5.4.2. Immune Pathways

Microbial antigens and metabolites that translocate across a compromised intestinal barrier activate systemic immune cells via pattern recognition receptors (particularly TLR4). Activated peripheral immune cells can then infiltrate the brain through a disrupted BBB, amplifying local neuroinflammation. This pathway is particularly relevant in VCI, where BBB dysfunction is a consistent feature across multiple disease subtypes [L2: Inaba et al. TLR4-KO; L1: correlational evidence from CSVD] [55].

#### 5.4.3. Neuroendocrine Pathways

The HPA axis and sympathetic nervous system are activated in response to cerebral ischemia/hypoxia, releasing corticotropin-releasing hormone and catecholamines that act on enteric glial cells and intestinal epithelial cells. This neuroendocrine response impairs mucus production and antimicrobial peptide secretion, thereby increasing intestinal paracellular permeability and perpetuating the “leaky gut” state [L2: Animal models of cerebral ischemia; L4 for human VCI].

This tripartite framework provides a more nuanced understanding of how gut–brain communication operates in VCI compared to a simple linear model, and highlights multiple potential intervention points for disrupting the pathological cycle.

## 6. Summary and Prospect

This review systematically outlines the core mechanisms by which intestinal mucosal barrier damage and gut dysbiosis drive neuroinflammation in VCI. Chronic cerebral hypoperfusion initiates cerebral ischemia/hypoxia, activating microglia, triggering a pro-inflammatory cascade (TNF-α, IL-1β, IL-6), and disrupting the BBB, culminating in neuronal loss, white matter lesions, and cognitive decline. Concurrently, a “leaky gut” permits translocation of microbial products (e.g., LPS) into the circulation, which activates systemic inflammation and further compromises the BBB, amplifying central neuroinflammation. Reduced protective SCFAs exacerbate this process. Critically, cerebral ischemia reciprocally impairs gut barrier function via neuroendocrine pathways (HPA axis and sympathetic activation), forming a self-sustaining vicious cycle: barrier damage → systemic inflammation → BBB disruption → neuroinflammation → neuronal damage → worsened ischemia → further barrier deterioration. Preclinical evidence supports that gut-targeted interventions (e.g., FMT, SCFA replenishment) can break this cycle and improve cognitive outcomes, offering promising translational avenues.

Despite these advances, several critical evidence gaps remain: (1) the field is dominated by animal and in vitro data, with a striking paucity of large-scale prospective human cohorts and randomized controlled trials; (2) the specific signaling pathways, key microbial species/metabolites, and their spatiotemporal dynamics in VCI remain poorly defined; (3) existing intervention studies suffer from small sample sizes, short follow-up durations, and a lack of objective response markers (e.g., neuroimaging, CSF/blood biomarkers) to validate central effects; (4) critical confounders—including genetic background, dietary habits, comorbidities (diabetes, hypertension), and concomitant medications—have not been systematically controlled; (5) donor–recipient heterogeneity, optimal therapeutic windows, and maintenance regimens for gut-targeted interventions remain entirely unresolved.

To address these gaps, future research must adopt rigorously designed, multi-center, randomized, placebo-controlled clinical trials with clearly defined VCI subtypes. Table 3 provides a comprehensive set of recommended design parameters—covering study population, intervention protocols, outcome measures (cognitive, vascular, gut-specific, and imaging biomarkers), microbiome analytical standards, and follow-up requirements—to guide the next generation of translational studies in this field.

Looking forward, the integration of multimodal imaging markers, fluid biomarkers, and detailed neuropsychological assessments—as outlined above—will enable the construction of a multidimensional VCI subtyping system. This will allow for precise identification of patient subgroups most likely to benefit from gut-targeted intervention strategies, thereby advancing VCI management from a “one-size-fits-all” approach toward a genuinely individualized precision medicine model. The ultimate goal is to translate these mechanistic insights into safer, more effective strategies for early warning, prevention, and disease modification in VCI.

## Figures and Tables

**Table 3:** Recommended design parameters for future clinical studies on gut barrier–neuroinflammation in VCI.

Parameter	Recommendation
Study Population	Clearly defined VCI subtype (preferably subcortical ischemic vascular disease or mixed AD/vascular pathology); baseline intestinal permeability status defined
Study Design	Multi-center, randomized, double-blind, placebo-controlled, parallel-group design
Donor/Intervention Selection	Standardized healthy donors or defined microbial consortia; characterize microbiome composition and metabolic profile
Intervention Regimen	Oral capsules, FMT, or specific prebiotics/synbiotics; define single-dose versus multi-dose schedules; consider antibiotic preconditioning
Control Arm	Placebo capsules or sham procedure
Cognitive Outcomes	MoCA, executive function tests (TMT-B), memory tests (RAVLT)
Vascular Outcomes	WMH quantification (Fazekas score), CBF assessment (ASL-MRI), BBB permeability (DCE-MRI)
Gut-Specific Outcomes	Fecal microbiome composition (shotgun metagenomics), intestinal permeability markers (zonulin), fecal calprotectin, SCFA quantification
Blood Biomarkers	LPS, IL-6, TNF-α, GFAP, NfL, P-tau181, Aβ42/40 ratio
Imaging Biomarkers	^18^F-FDG PET or ASL-MRI (brain metabolism/perfusion); TSPO-PET (neuroinflammation, optional)
Microbiome Analysis	Shotgun metagenomics (superior to 16S rRNA) + targeted/untargeted metabolomics
Safety Monitoring	Adverse event grading (CTCAE criteria); infection risk surveillance
Follow-up Duration	Minimum 12 months; assessments every 3–6 months
Statistical Analysis	Intention-to-treat principle; mixed-effects models for repeated measures; correction for multiple comparisons

## Data Availability

No new data were created or analyzed in this study. Data sharing is not applicable to this article.

## Abbreviations

The following abbreviations are used in this manuscript:

AD	Alzheimer’s disease
Aβ	Amyloid beta
AMPK	AMP-activated protein kinase
APP	Amyloid Precursor Protein
ATP	Adenosine triphosphate
BBB	Blood–Brain Barrier
CADASIL	cerebral arteriopathy with subcortical infarcts and leukoencephalopathy
CBF	Cerebral blood flow
CCH	Chronic Cerebral Hypoperfusion
CRH	Corticotropin-releasing hormone
CSVD	Cerebral small vessel disease
ERK/MAPK	Extracellular signal-regulated kinase/Mitogen-activated protein kinase
FMT	Fecal microbiota transplantation
HPA	Hypothalamic-pituitary-adrenal (axis)
IL-6	Interleukin-6
Lp-PLA2	Lipoprotein-associated phospholipase A2
LPS	Lipopolysaccharide
MMP-9	Matrix metalloproteinase-9
mTORC1	mechanistic target of rapamycin complex 1
NF-κB	Nuclear factor-kappa B
NLRP3	NOD-like receptor family pyrin domain containing 3
PSCI	Post-stroke cognitive impairment
PVS	Perivascular spaces
sIgA	secretory immunoglobulin A
SCFAs	short-chain fatty acids
TCM	Traditional Chinese medicine
TJ	Tight Junction
TLR4	Toll-like receptor 4
TNF-α	Tumor necrosis factor-alpha
VaD	Vascular dementia
VCI	Vascular cognitive impairment
VISTA	V-domain immunoglobulin suppressor of T cell activation
